# Understanding Melanocyte Stem Cells for Disease Modeling and Regenerative Medicine Applications

**DOI:** 10.3390/ijms161226207

**Published:** 2015-12-21

**Authors:** Amber N. Mull, Ashwini Zolekar, Yu-Chieh Wang

**Affiliations:** Department of Pharmaceutical Sciences, University of North Texas Health Science Center, 3500 Camp Bowie Boulevard, Fort Worth, TX 76107, USA; amber.mull@unthsc.edu (A.N.M.); ashwini.zolekar@unthsc.edu (A.Z.)

**Keywords:** melanocytes, melanocyte stem cells, epidermis, pluripotent stem cells

## Abstract

Melanocytes in the skin play an indispensable role in the pigmentation of skin and its appendages. It is well known that the embryonic origin of melanocytes is neural crest cells. In adult skin, functional melanocytes are continuously repopulated by the differentiation of melanocyte stem cells (McSCs) residing in the epidermis of the skin. Many preceding studies have led to significant discoveries regarding the cellular and molecular characteristics of this unique stem cell population. The alteration of McSCs has been also implicated in several skin abnormalities and disease conditions. To date, our knowledge of McSCs largely comes from studying the stem cell niche of mouse hair follicles. Suggested by several anatomical differences between mouse and human skin, there could be distinct features associated with mouse and human McSCs as well as their niches in the skin. Recent advances in human pluripotent stem cell (hPSC) research have provided us with useful tools to potentially acquire a substantial amount of human McSCs and functional melanocytes for research and regenerative medicine applications. This review highlights recent studies and progress involved in understanding the development of cutaneous melanocytes and the regulation of McSCs.

## 1. Introduction

As a vital organ in the body, the skin forms a physical barrier between internal organs and the environment. It has many important functions in maintaining the homeostasis of body temperature and the balance of body fluid. In addition, the skin serves as a primary defensive mechanism against pathogens and environmental assaults. Anatomically, human skin consists of the epidermis and dermis. Many cell types are located in the dermis including dermal fibroblasts, neuronal cells, smooth muscle cells, endothelial cells and immune cells. The epidermis is primarily made of keratinocytes and melanocytes. In addition, Langerhans cells (a type of resident innate immune cells) can be found between keratinocytes. Several skin appendages such as hair follicles and sebaceous glands also exist in the epidermal layer of skin. There is a team of stem cells that serve as a reservoir to generate new cells and sustain the proliferative feature and normal function of the epidermis. Located in hair follicles and the basal layer of the interfollicular epidermis, human cutaneous melanocytes produce and distribute melanin, which leads to hair and skin pigmentation, absorbs ultraviolet (UV) light, and protects the skin from UV radiation-induced damage. The cellular origin and development of melanocytes during embryogenesis are extensively investigated. Many studies have also identified the role of melanocyte stem cells (McSCs) in the maintenance of melanocyte populations in adult skin. In this review article, we discuss recent progress towards understanding the development of cutaneous melanocytes, the characteristics of McSCs, and how McSCs may contribute to normal and pathological melanogenesis.

## 2. The Development of Melanocytic Lineage during Embryogenesis

**Figure 1:**
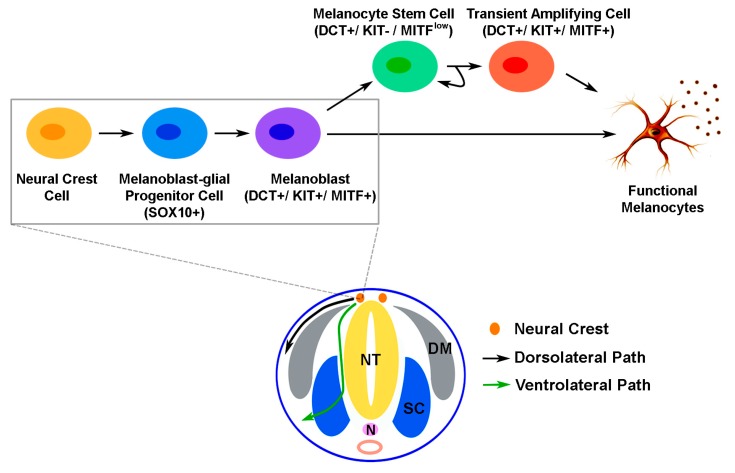
A schematic illustration of the migratory paths of neural crest cells and melanocyte development in a developing mammalian embryo. Cells that migrate from the trunk neural crest through the developing embryo may take a dorsolateral or ventrolateral path. Melanocytic differentiation from trunk neural crest cells begins with the specification of neural crest cells into melanoblast-glial progenitor cells which express SRY (sex determining region Y)-box 10 (SOX10). These progenitor cells are then committed into melanoblasts with the expression of dopachrome tautomerase (DCT), tyrosine protein kinase KIT (KIT) and microphthalmia-associated transcription factor (MITF). The melanoblast progenitor cells typically migrate along the dorsolateral path between the epidermis and dermomyotome. During embryogenesis, melanoblasts can move into embryonic hair follicles where some melanoblasts continue to differentiate into functional melanocytes that produce melanin and participate in the initial hair cycle. Subsets of melanoblasts become melanocyte stem cells (McSCs), which have the capacity of self-renewal. McSCs remain quiescent until activated in the next hair cycle, resulting in transient amplifying cells and their subsequent differentiation into functional melanocytes. *NT:* neural tube, *N:* notochord, *DM:* dermomyotome, *SC:* sclerotome.

The development of melanocytic lineage has long been of special interest to developmental biologists. This particular cell lineage is derived from neural crest cells (NCCs) which are formed during the neurulation process in a developing embryo. NCCs are highly mobile and capable of giving rise to multiple cell lineages including melanocytes [1,2]. Based on the anteroposterior position of NCCs in the embryo, they can be categorized into five subtypes including cranial, vagal, sacral, trunk and cardiac crest cells. Although most NCC populations appear to be capable of committing to the melanocytic lineage, it is believed that most melanocytes in skin originally arise from trunk crest cells [3,4]. NCCs and their derivatives in the trunk region can migrate through the developing embryo via dorsolateral or ventrolateral routes (Figure 1). In mammalians, melanocytic lineage commitment starts from the specification of NCCs with the expression of SRY (sex determining region Y)-box 10 (SOX10) in the trunk region to form melanoblast-glial bipotent progenitor cells. These bipotent progenitor cells are further specified with the activation of microphthalmia-associated transcription factor (MITF) to become melanoblasts positive for MITF, dopachrome tautomerase (DCT) and tyrosine protein kinase KIT (KIT) (Figure 1). These cells are proliferative, capable of migrating over a long distance in the dorsolateral route [3], and can terminally differentiate into melanin-producing melanocytes.

**Figure 2:**
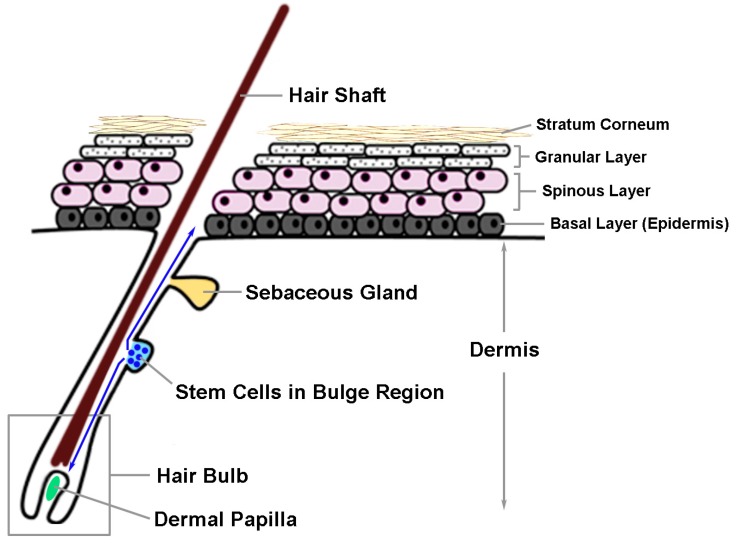
The anatomical structure of a hair follicle in mammalian skin. Normal skin is composed of the epidermis and dermis. In the epidermis, several skin appendages such as hair follicles and sebaceous glands can be found. The bulge region of hair follicles is a well-known niche for stem cells in the skin. Hair follicle stem cells (HFSCs) and melanocyte stem cells (McSCs) reside in this niche. Usually, HFSCs and McSCs migrate toward the base of hair follicles and differentiate into keratinocytes and melanocytes to assemble pigmented hairs during the anagen of a hair cycle. In certain conditions, the niched McSCs in hair follicles may migrate upward and differentiate into melanocytes at the basal layer of the epidermis. This bi-directional migratory path of McSCs in hair follicles is indicated by the blue arrows.

On the other hand, NCCs that migrate in the ventrolateral route typically take on a neuronal (e.g., sensory or sympathetic nerve), glial (e.g*.*, Schwann cell precursors) or endoneural fibroblast fate [3]. Although cells migrating in the dorsolateral route appear to exclusively adopt a melanocytic fate [3], additional evidence has suggested that a subpopulation of adult melanocytes in the trunk region may arise from melanoblast-glial bipotent progenitor cells which have migrated in the ventrolateral route down to the developing nerve tissue. Observed in both chicken and mouse embryos, there is a loss of melanoblasts from the dorsolateral path between E10.5/HH24 and E11.5/HH27 (mouse/chicken) [5]. These lost melanoblasts are replaced by a second wave of melanoblasts that arise from cells in close proximity to the distal, ventral ramus of the spinal nerve, which is blocked if the ventrolateral migration is suppressed in chicken embryos [5]. In mice with Schwann cell precursors that are lineage-labeled using *Plp-CreErt2*, the origin of 65% of melanocytes in hair follicles can be traced back to this precursor population [5]. Although it has not been examined in humans, these findings support the likelihood of growing nerve as a potential niche that contains progenitor cells and signaling for human melanocyte development *in vivo*. After being incorporated into the embryonic hair follicles, some melanoblasts differentiate into melanocytes and immediately contribute to the color of hair generated in the first hair cycle. Meanwhile, the expression of MITF and KIT can be downregulated in certain DCT-positive melanoblasts to form melanocyte stem cells (McSCs) that reside in the hair follicle bulge region (Figure 2). How this process is modulated still remains elusive; however, it is known that this unique stem cell population plays an important role in the replenishment of terminally differentiated melanocytes in each new hair cycle to generate pigmented hair [6].

Among many factors involved in the development of melanocytes, MITF appears to be the pivotal regulator that determines melanocyte identity. Indeed, mutations in the human *MITF* gene can lead to Waardenburg syndrome type 2 (WS2) and Tietz syndrome which are dominantly inherited syndromes with the disease phenotype of hypopigmentation and hearing loss [7,8]. Many factors are involved in the regulation of MITF expression during the specification and development of melanocytic lineage. As a growth factor, wingless-type MMTV integration site family member 3A (WNT3A) induces the expression of Mitf in cultured mouse melanocytes and melanoblast formation in avian NCCs [9,10,11], suggesting that WNT3A is critical for the initiation of melanocyte differentiation. In addition to WNT3A, growth factors such as stem cell factor (SCF, KIT ligand), endothelins, ephrins and bone morphogenetic protein 4 (BMP4) have been also implicated with the regulation of melanocyte development [12,13,14]. Although signaling through receptor tyrosin kinase KIT does not seem required for melanocytic lineage specification, it has been shown that KIT and KIT ligand are crucial for both the survival and migration of melanoblasts [15,16,17]. The KIT-mediated survival and migration of melanoblasts, however, appear to rely on different mechanisms downstream of KIT. Using mouse models, Wehrle-Haller *et al.* demonstrated that the KIT ligand-induced migration of melanoblasts, unlike the survival of melanoblasts, does not require the activation of mitogen-activated protein kinase (MAPK) signaling [18].

Along the process of melanocyte differentiation, the expression of MITF is intricately regulated by multiple transcription factors. For example, PAX3 and SOX10 have been known for their synergistic regulation of *MITF* gene transactivation [19,20,21]. The phenotypes of *PAX3* and *SOX10* gene mutations in mice, however, indicate that these two transcription factors also govern the development of neural cells differentiated from NCCs [22]. Thus, other mechanisms that control the cell fate switch between neural and melanocytic linages are supposed to exist in NCCs. Additional studies have revealed that FOXD3 and SOX2 are responsible for the suppression of *MITF* gene expression activated by PAX3 and SOX10 in NCCs [23,24], by which the differentiation of NCCs is biased toward the neural lineage. The downregulation of FOXD3 and SOX2 in NCC-derived, melanoblast-glial bipotent progenitor cells is therefore considered crucial for their efficient commitment to the melanocytic lineage. Interestingly, the expression of MITF in the cells causes a negative feedback regulation on FOXD3 and SOX2. It has been reported, at least in chicken embryos, that the ectopic expression of MITF in NCCs committed to the glial cell fate can lead to the downregulation of FOXD3 and SOX2 [23,25], attesting to the role of MITF in the reinforcement of melanocytic fate that it drives during melanogenesis in NCCs. Evidence supporting the indispensable role of MITF in melanogenesis and molecular mechanisms that regulate MITF expression in cells has been comprehensively reviewed by Mort *et al.* [1] as well.

## 3. Melanocyte Stem Cells (McSCs) in Hair Follicles

To date, McSCs in hair follicles have been studied most extensively in mouse models. The bulge and bulb (secondary hair germ) regions of hair follicles contain different types of stem cells. In a normal hair follicle, hair follicle stem cells (HFSCs) and McSCs are frequently found in these stem cell niches. The cells in the secondary hair germ are derived from bulge cells during the development of hair follicles and are considered the closely related extension of bulge cells [26]. Although certain differences exist between bulge and secondary hair germ cells, secondary hair germ cells highly resemble the bulge cells on a functional level [26]. Interestingly, almost all bulge cells that undergo apoptosis after depilation are soon repopulated by residual proliferating cells in the secondary hair germ at the beginning of anagen for hair regrowth [27]. These newly formed bulge cells later regain bulge-specific markers (e.g., CD34, Nfatc1 and S100A4) [27], further attesting to the dynamic interaction and common origin of stem cell populations in the bulge and bulb niches of an active hair follicle.

Both HFSCs and McSCs remain quiescent during the telogen phase of a hair cycle. The niched McSCs are amelanotic and unaffected by the deprivation of KIT signaling [6]. With the initiation of a new hair cycle, these two stem cell populations are activated primarily due to enhanced wingless (Wnt) signaling and reduced transforming growth factor β (TGFβ) signaling, which crosstalk with signaling molecules provided by dermal papilla at the base of a hair follicle [28]. At this stage, both HFSCs and McSCs contain nuclear β-catenin [28], highlighting the significance of the canonical Wnt signaling pathway in the coordination of HFSCs and McSCs for proliferation and differentiation. Interestingly, the deficiency of Col17a1 and Nf1b in HFSCs can lead to the premature differentiation of McSCs in mouse hair follicles [29], suggesting that the synchrony of HFSCs and McSCs also depends on their interactions. HFSCs can also release Wnt, TGFβ and endothelin proteins that are critical for the activation, proliferation and maintenance of McSCs [28,29,30], forming another possible mechanism for HFSCs to coordinate with McSCs in a shared niche. Whether and how McSCs may reciprocally regulate HFSCs in the niche is currently unclear and requires future studies. As the result of orchestrated behaviors of these stem cell populations, hair matrix keratinocytes that contain melanocyte-transferred melanin are formed and assembled into pigmented hair shafts.

In response to wounding or UV irradiation, follicular McSCs can exit the stem cell niche before their initial cell division, migrate toward the basal layer of the epidermis in a melanocortin 1 receptor (Mc1r)-dependent manner, and differentiate into functional epidermal melanocytes [31]. This upward migration behavior of McSCs in hair follicles due to environmental stimuli provides a mechanistic rationale for developing therapeutic approaches to treat skin hypopigmentation disorders by manipulating this stem cell population. In fact, narrow-band ultraviolet B (UVB) exposure has been used to treat patients with vitiligo and leads to follicular repigmentation of depigmented skin by causing the proliferation, migration and differentiation of McSCs [32,33].

In addition to HFSCs and derma papilla cells, other cell types including immune cells, neuronal cells and endothelial cells may influence McSCs in hair follicles. Skin-resident immune cells, dendritic epidermal T cells (DETCs) and Langerhans cells can be found in the mouse epidermis, whereas dendritic cells, mast cells, macrophages and T cells are often located in the dermis [34,35]. Similar to immune cells in the circulatory system, skin-resident immune cells can release cytokines and growth factors. Several of these factors are known for their roles in the regulation of epidermal physiology. For example, fibroblast growth factor 7 (Fgf7), Fgf10 and insulin-like growth factor 1 (Igf1) released by DETCs have been shown critical for the activation epidermal cells due to the compromised epidermal barrier [36,37]. Fgf9 released by dermal T cells also promote Wnt signaling activation to induce hair follicle morphogenesis in wounded epidermis [38]. These findings suggest that skin-resident immune cells such as DETCs and dermal T cells may play an important role in modulating the stem cell niche in hair follicles and the behavior of HFSCs and McSCs. Besides immune cells, sensory nerve endings and capillary networks in the skin can be located in close proximity to the bulge region of hair follicles. Some evidence has suggested that skin- and hair bulge-derived signaling molecules can affect the process of innervation and vascularization in skin [39,40,41]. Although additional studies are required to clearly define how the signaling crosstalk occurs between nerve, blood vessels and hair follicles, it is possible that secretory factors of sensory neurons and endothelial cells can help to shape the stem cell niche and have an impact on the cell fate of HFSCs and McSCs. Interestingly, Brownell *et al.* have shown that Sonic hedgehog (Shh) generated by sensory nerves can signal to the upper bulge region of hair follicles and regulate the epidermal stem cell fate of hair bulge cells [42].

The discoveries discussed above have shed light on many potential targets to modulate McSCs for research and clinical applications. Additional study of the interactions between hair follicle McSCs and other cell types in specific microenvironment and timing may provide new insights into how different cell lineages coordinate with each other to ensure the formation of pigmented hair and skin.

## 4. Interfollicular McSCs

The comparison between mouse and human skin tissue has revealed several distinct features associated with each species. Murine cutaneous melanocytes are almost exclusively located in hair follicles and the dermis and not in the epidermis (except for few areas such as tail and paw skin where epidermal melanocytes can be found). In contrast, human skin contains large interfollicular areas that are populated with melanocytes at the basal layer of the epidermis. Thus, melanocytes and McSCs in human skin could behave differently from those in mouse skin. In addition, the anatomical feature of melanocytes in human skin suggests that there may be a unique stem cell reservoir independent of the McSCs in hair follicles to sustain the melanocyte population in the interfollicular epidermis. Although this putative “interfollicular epidermal McSC population” has not been clearly defined and identified (to our knowledge), a recent study using mouse tail skin that lacks appendages revealed that the appendage-free mouse tail skin maintain a stable melanocyte population, including a low frequency of amelanotic melanocytes [43]. This discovery provides experimental evidence supporting the idea that interfollicular epidermal melanocytes are sustained by mechanisms other than McSCs in hair follicles.

Unlike the production of KIT ligand found in both the human epidermis (keratinocytes) and dermis (fibroblasts and endothelial cells) [44,45], the expression of SCF in postnatal murine skin is primarily restricted to the dermis and hair follicles and missing in epidermal keratinocytes [46]. The ectopic expression of SCF in mouse epidermal keratinocytes leads to the maintenance of melanocytes in the interfollicular epidermis and postnatal skin hyperpigmentation [47,48], highlighting the critical role of epidermal KIT ligand in the regulation of interfollicular melanocytes. Further analysis in transgenic and wild-type mice reveals that DCT-positive melanoblasts are found in the basal cell layer of the oral epithelium and the epidermal skin of the 16.5 dpc transgenic mouse embryos with epidermal SCF expression, but they are not found in the corresponding part of the wild-type litters [48]. This observation suggests that the progenitor cells of melanocytes in the 16.5 dpc transgenic mouse embryos have arrived at the interfollicular epidermal areas that do not typically contain melanocytes in mice. Thus, the presence of epidermal KIT ligand is likely to be a key modulatory mechanism for the distribution and behavior of McSCs in the mammalian interfollicular epidermis and is ultimately responsible for the maintenance of melanocytes in the basal layer of the human epidermis and postnatal skin pigmentation.

Interestingly, Li *et al.* have isolated dermal stem cells from human skin that are capable of differentiating into functional melanocytes [49]. In addition, a melanocytic progenitor niche in sweat glands and interfollicular Axin2-expressing epidermal stem cells have been recently reported [50,51]. Although these stem cell niches may all contribute to melanocytic homeostasis in the interfollicular epidermis, additional studies are needed to test whether they are locations where the definitive interfollicular epidermal McSCs reside.

## 5. McSCs in Phenotypic Abnormalities and Pathological Conditions

The alteration of McSCs due to various mechanisms has been implicated in several phenotypic abnormalities and pathological conditions. Associated with hair greying, a gradual decline in the number of McSCs in hair follicles during the aging process has been noticed in both mice and humans [52]. Repetitive ablation of melanocytes in zebra fish can eventually lead to depletion of McSCs and loss of hypodermal melanocyte stripes [53]. These findings indicate that McSCs are a specified and limited cell population that can be exhausted even though they seem to have the potential for self-renewal in the adult body. In addition to the physiological aging process, environmental stresses and stimuli such as wounding and exposure to radiation and chemicals can also lead to the depletion of McSCs and hair depigmentation [31,54,55].

As one of the hypopigmentation skin disorders, the clinical presentation of vitiligo is usually characterized by acquired, progressive, circumscribed loss of pigmentation in patients' skin and hair, with a total absence of melanocytes microscopically [56]. Although the etiology of vitiligo is not completely understood, it is generally believed that autoimmune responses that target melanocytes play a major role in the pathogenesis of vitiligo [57]. Interestingly, this anti-melanocyte autoimmunity appears to spare McSCs in the bulge region of hair follicles within vitiligo skin lesions, evidenced by the UV-induced follicular repigmentation of the lesions [32,33]. This "sparing effect" is potentially due to the amelanotic feature of McSCs, which may make McSCs less antigenic compared to melanin-producing melanocytes. In addition, hair follicle cells including McSCs may generate cytokines that repel or suppress immune cells [58]. This finding also suggests that McSCs possibly contribute to the modulation of immune cells and skin inflammation after wounding.

McSCs are also considered a possible cell origin for melanoma. As the leading cause of skin cancer-relevant death, melanoma is notoriously known for its ability to metastasize [59]. The propensity of melanoma cells for distant metastasis [60] closely resembles the migratory feature of melanocyte progenitors such as neural crest cells, melanoblasts and McSCs. Similar to the cancer stem cells in acute myeloid leukemia [61], McSCs may be the target of oncogenic transformation and give rise to melanoma-initiating cells. Although the evidence to definitively prove the correlation between the abnormality of McSCs and melanoma formation is still absent, several groups are actively working on relevant topics in the field.

## 6. Melanocyte Differentiation in Pluripotent Stem Cells

Pluripotent stem cells (PSCs), including embryonic stem cells (ESCs) and induced pluripotent stem cells (iPSCs), can proliferate indefinitely and give rise to virtually any type of embryonic and adult cells upon receiving differentiation cues. Because of their tremendously diverse differentiation capacity, pluripotent stem cells have become a powerful tool to facilitate basic research and regenerative medicine. Several groups including ours have reported the successful differentiation of human and mouse PSCs (hPSCs and mPSCs) toward the melanocytic lineage [62,63,64,65,66,67,68]. By differentiating cultured hPSCs into functional melanocytes, we are likely to recapitulate the normal developmental process of human melanocytes in an *in vitro* condition. Mica *et al.* have shown compelling data in support of the presence of DCT+/KIT+/MITF+ melanoblasts in the process of directed melanocyte differentiation [68]. Although additional characterizations are needed to show that DCT+/KIT−/MITF^low^ McSCs can be efficiently acquired and maintained using the existing differentiation protocols, the derivation of McSCs from hPSCs is theoretically achievable. Since hPSC culture can be easily scaled up, hPSCs represent a valuable source to generate a large number of human McSCs and melanocytes for research and potential clinical applications.

Previously, techniques to isolate and culture melanoblasts from mouse and human skin were established [69,70,71,72,73]. Researchers also attempted to isolate and culture McSCs from hair follicles. Some success has been obtained in culturing and expanding hair follicle McSCs isolated from mouse skin tissue in an *in vitro* condition [74]. However, whether a similar isolation and culture approach is applicable to human hair follicle McSCs still remains to be determined. The integration of hPSC differentiation techniques, existing approaches for culturing McSCs and melanoblasts isolated from skin tissue, and the knowledge of stem cell niches in skin may lead to the development of novel platforms for studying human melanogenesis in normal and pathological conditions.

## 7. Modeling Pathological Melanogenesis Using Pluripotent Stem Cell Techniques

Human PSCs are a powerful tool to study and potentially treat human disease. The invention of human iPSC (hiPSC) techniques [75] together with numerous relevant studies that use patient-derived iPSCs and their differentiated derivatives to recapitulate tissue-specific disease phenotypes have revolutionized our concept on how human disease can be modeled and studied. By subjecting patient-derived iPSCs that carry genetic abnormalities associated with pathological melanogenesis to melanocyte differentiation, we are likely to acquire human pathogenic melanocytes which accurately present disease phenotypes that would not show in other types of human cells or melanocytes of other animal species. Indeed, Mica *et al.* have reproduced the ultrastructural features of pigmentation defects in the melanocytic differentiated derivatives of Hermansky-Pudlak syndrome and Chediak-Higashi syndrome patient-derived iPSCs [68]. Another recent study has also used this research approach to identify a novel role of the *NF1* gene in the regulation of cellular senescence in melanocytic lineage [76]. With further adaptation, the melanocytic differentiation of hPSCs may be used to dissect the oncogenic development of melanoma.

In addition to the melanocytes differentiated from patient-derived iPSCs, the reprogramming of human melanocytes to obtain cells with the features of hPSCs or multipotent stem cells may form another unique platform to study melanocyte-associated diseases. Since SOX2 is dispensable for reprogramming human melanocytes into hiPSCs [77], melanocytes could be considered a somatic cell type that has less reprogramming barriers. It is also known that melanoma cells can express certain stem cell markers and differentiate in response to embryonic microenvironments (reviewed in [78] and [79], respectively), suggesting the oncogenic reprogramming or dedifferentiation of melanocytes as a potential mechanism that leads to the formation of melanoma cells. Thus, melanocytes partially reprogrammed using reprogramming factors for the generation of hiPSCs are likely to mimic the heterogeneous melanoma cells in a tumor. Future studies are required for testing this hypothesis and the feasibility of using partially reprogrammed melanocytes to model melanoma.

## 8. Conclusions

McSCs play important roles in the maintenance of melanocyte populations in normal adult skin and its appendages. Studies on McSCs have helped to dissect molecular mechanisms underlying normal melanocyte development as well as melanocyte-related diseases such as vitiligo and melanoma. Although research efforts in the past have also provided us with important information regarding the location, origin and markers of McSCs in human skin, the complete characterization of these cells has yet to be established. Emerging evidence suggests that alterations of McSCs may be frequently seen in a variety of human melanocyte-associated physiological abnormalities and pathological conditions. Recent advances in hiPSC research have offered exciting opportunities and unique platforms to better examine human McSCs and the development of human melanocytes. It is now possible to obtain a sufficient amount of patient-specific melanocytes or McSCs by the differentiation of hiPSCs for disease modeling and potential therapeutic purposes. We believe that the integration of rodent models, hPSC differentiation and cell reprogramming approaches in future studies for understanding McSCs shall lead to novel and significant insights that can ultimately benefit patients with different melanocyte-related diseases.
